# Scaling behaviours in the growth of networked systems and their geometric origins

**DOI:** 10.1038/srep09767

**Published:** 2015-04-29

**Authors:** Jiang Zhang, Xintong Li, Xinran Wang, Wen-Xu Wang, Lingfei Wu

**Affiliations:** 1 School of Systems Science, Beijing Normal University; 2 College of Resources Science and Technology, Beijing Normal University; 3 School of Human Evolution and Social Change, Arizona State University; 4 Center for the Study of Institutional Diversity, Arizona State University

## Abstract

Two classes of scaling behaviours, namely the super-linear scaling of links or activities, and the sub-linear scaling of area, diversity, or time elapsed with respect to size have been found to prevail in the growth of complex networked systems. Despite some pioneering modelling approaches proposed for specific systems, whether there exists some general mechanisms that account for the origins of such scaling behaviours in different contexts, especially in socioeconomic systems, remains an open question. We address this problem by introducing a geometric network model without free parameter, finding that both super-linear and sub-linear scaling behaviours can be simultaneously reproduced and that the scaling exponents are exclusively determined by the dimension of the Euclidean space in which the network is embedded. We implement some realistic extensions to the basic model to offer more accurate predictions for cities of various scaling behaviours and the Zipf distribution reported in the literature and observed in our empirical studies. All of the empirical results can be precisely recovered by our model with analytical predictions of all major properties. By virtue of these general findings concerning scaling behaviour, our models with simple mechanisms gain new insights into the evolution and development of complex networked systems.

Discovered quite recently, but with roots that go back to decades ago in biology and ecology^1^,^2^,^3^,^4^, the scaling behaviour is generally shared by a variety of complex networked systems, such as cities^5^,^6^,^7^,^8^, online communities^9^,^10^,^11^, and complex networks^12^. The scaling behaviours in the form 

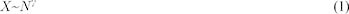

 capture the fact that some macroscopic variables 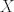 scale with the system size 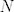 , where 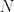 represents the number of entities in a networked system. Based on empirical observations of the exponent 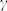 , two categories, super-linear and sub-linear scaling behaviours, have been identified.

For instance, in cities, if 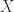 represents the gross domestic product (GDP), the total wage, or the number of crimes, and 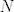 is the population of a city, a super-linear scaling with 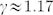 5,6,7,13 is found. 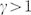 indicates that as a city grows, the total number of interactions increases at a faster rate, leading to more wealth and innovation per capita, but also with more crime and pollution as side effects. In online communities, 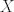 represents the total number of activities of users, such as clicks, micro-blogs and tags^9^,^10^,^11^, and 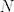 is the total number of active users. 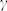 lies in the range [1.17-1.48]. The super-linear scaling behaviours have also been observed in complex networks, but in this context, it is termed accelerating growth^12^,^14^ or densification^15^, and is characterized by a higher generating rate for links than for nodes during network growth. This phenomenon is found in scientific collaborations^16^, citation network^15^, Internet autonomous system^15^,^17^, food webs^18^, and neural networks^19^.

Sub-linear scaling as another type of scaling behaviour with exponent smaller than 1 is also prevalent in complex networked systems. For instance, the area and road volume of a city are found to scale sub-linearly with its population^5^. In online communities, the number of distinct tags scales sub-linearly with the system size^9^. In information retrieval or in linguistics, the general Heap's law captures a sub-linear scaling between the number of distinct words and the total number of words^20^,^21^. In ecological communities, the diversity of higher taxa scales sub-linearly with the number of species^22^. The sub-linear scaling is also found in river networks^23^, various combinatorial systems^24^ and etc.

Although some effort has been dedicated to explaining the scaling behaviours based on network approaches^2^,^3^,^25^, forest-fire model^15^, random walks^9^, Kronecker graphs^26^ and the recently developed city model^27^, the origins of the scaling behaviours have not been fully understood yet. In particular, the general findings in diverse systems prompts us to wonder if there exists some simple but universal underlying mechanism that accounts for both classes of scaling behaviours. In this paper, we propose a geometrical network model to address this fundamental problem. Partially inspired by the hyperbolic space model for construction of scale-free networks^28^ and the hidden geometry of complex networks^29^, we propose a spatial-constrained attachment (SCA) model to uncover the origins of both super-linear and sub-linear scaling behaviours in geometric space of arbitrary dimension. The self-organized phenomena produced by our SCA model are in good agreement with a variety of empirical findings, including the scaling behaviours governing the entire number of links, the time elapsed, and the volume versus the system size measured as the number of nodes in the network. The simple mechanism of SCA allows us to derive analytical results for all critical network properties, such as the accelerating growth, degree distributions of nodes, and the clustering coefficient. We also slightly modify the model by considering some realistic restrictions to better mimic real situations. We apply our model to online communities, a citation network and nighttime light clusters, finding that the empirical observations from these cases, including the scaling behaviours, Zipf size distribution^30^, and aggregation patterns, can be quantitatively reproduced.

## Results

### The Model

**Figure 1:**
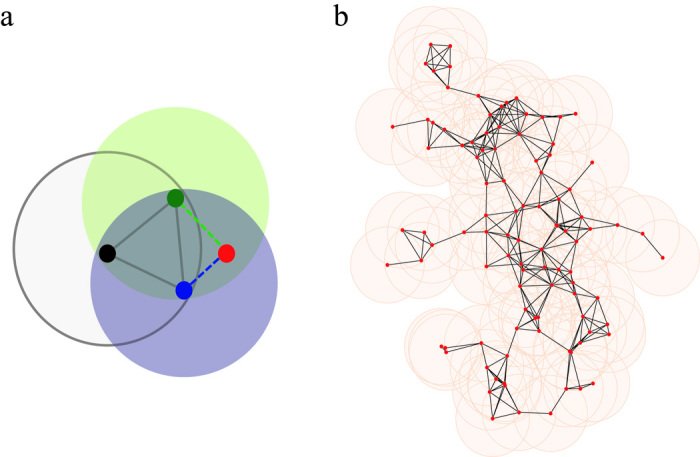
An illustration of the basic model in *d* = 2-dimensional space. (a) The filled disks represent existing nodes, the red disk represents a newly added node that will survive. The dark lines represent existing links, and the dashed lines represent the newly added links. The shaded areas represent the interaction regions of existing nodes. (b) A simulation of the basic model after 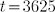 steps and 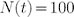 nodes. The shaded area represents 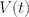 in two-dimensional space. In all simulations 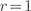 .

Our model assumes that a geometric graph grows in an 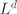 hypercube embedded in 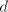 -dimensional Euclidean space^31^,^32^,^33^ according to the SCA mechanism, where 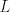 is the linear size of the hypercube and 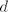 is the spatial dimension. Suppose that initially, a single node is present at the center of the hypercube as the seed of the growing graph. At each time step 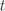 , one new node 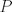 is generated, and its coordinates (location) 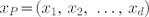 are randomly selected with equal probability of being located anywhere throughout the hypercube. However, 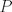 can survive only if 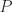 matches with at least one of the existing node 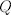 with location 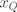 , such that 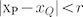 , where 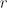 is a given parameter and 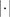 is the Euclidean distance. The surviving node 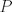 is then attached to all the existing nodes that belong to the 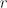 -radius ball of 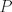 . In other words, interactions can be built exclusively within a certain area, limited by parameter 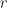 . This process is repeated until a network of the desired size is achieved (see Fig. 1). The geometric network grows at increasing speed in the sense that a newly added node is more likely to attach to existing nodes as the network volume increases. The central area around the seed is of higher density than the other areas because “dense gets denser.”

Although the evolution of complex networked systems in different disciplines is affected by many factors, our model captures some underlying mechanisms that are shared among a variety of systems with spatial constraint and play significant roles in the rising of scaling behaviours. Here, space can be classified into real geographical or physical spaces and, abstract spaces. Cities^27^, Internet autonomous systems^17^ and brain^19^ are subject to the former and, similarity spaces (scientific collaborations, citation networks, online communities)^28^,^34^,^16^,^15^, semantic spaces (languages, tagging systems)^9^ and niche spaces (ecological communities, food webs)^35^ are subject to the latter. In our model, the SCA mechanism uncovers the fact that the survival probability of a new node and its contribution to the growth of interactions are significantly determined by its closeness to others that can be measured by its relative location in a space. Indeed, the establishment of many kinds of interactions is strongly affected by the closeness among entities in a certain space, such as scientific collaborations, citations, social ties in cities and online communities, connections of routers, neuronal connections, and co-occurrence of tags. In general, two closer entities are of higher probability to build a connection between them, resulting from either the cost of establishing connections in physical spaces, or similarity induced connections in abstract spaces. On the other hand, the location of a new node may be determined by a number of factors, precluding us from specifying the exact location of every newly generated node. In this regard, it is reasonable to assume that new nodes are randomly located in the statistical point of view. Both closeness induced connections and random birth of new nodes can be captured by our SCA mechanism. Although it is not sufficiently concrete to model the evolution of every aspect of real networked systems, the SCA mechanism in our model is common in many real systems, accounting for the broad implications of our model in offering deeper understanding of scaling behaviours.

### Analytic Results

We derive the analytical results of our model in thermodynamic limit 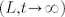 . As 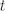 approaches infinity, the spatial shape of the network can be approximated by a symmetrical 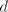 -dimensional ball. The radius 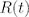 (which is defined as the maximum distance between any existing nodes and the seed (center)) of the ball at time 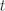 is linearly dependent on 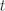 : 

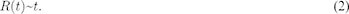

It is valid because that the average time between two updates to the nodes at the perimeter of the 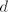 -dimensional ball is approximately ~ 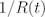 and there are approximately ~ 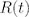 positions at the perimeter to be updated; thus the average speed at which the radius increases is a constant (~ 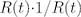 , see Supplementary Information Section 1). Then the total volume 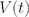 that is filled by all 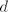 -dimensional balls with radius 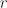 can be approximated by 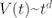 . Suppose that the density of nodes at any location with spherical coordinate 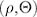 is 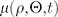 , where 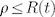 is the spherical radius and 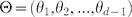 is the vector of spherical angles. Then after time step 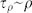 , the density 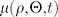 within the infinitesimal space 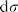 starts to become non-zero and increases with the constant rate 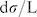 until 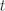 . Thus, the total number of nodes in the infinitesimal volume 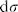 is 

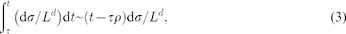

yielding 

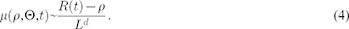

The density of nodes at radius 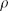 is 

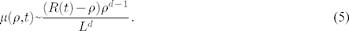

 Consequently, the total number of nodes within the radius 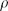 is approximated by 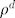 if 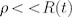 , in agreement with the fractal dimension 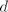 of the network in the thermodynamic limit^36^,^37^. Finally, the total number of nodes in the whole network can be calculated by 

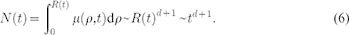

Note that each node at location 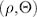 at time 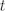 is connected to 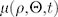 neighbors on average, and there are 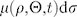 nodes in total in the infinitesimal space 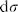 , meaning that the total number of links in 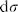 is 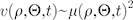 . Thus, the total number of links in the entire network is 

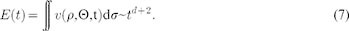

We can reformulate all the variables as functions of the number of nodes at 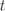 to obtain the scaling behaviours. The first scaling, between the total number of edges and nodes, can be obtained by eliminating 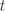 in both Eqs. (6) and (7): 

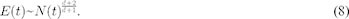

 This formula is consistent with Eq. (1) if we consider the total number of interactions to correspond to the number of links and the population is proportional to the number of nodes in a system. We see that the exponent 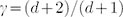 depends exclusively on the dimension of the space in which the spatial network is embedded. Another interesting scaling behaviour, between the volume and the size of the network, can be derived by Eq. (6): 

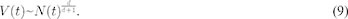

**Figure 2:**
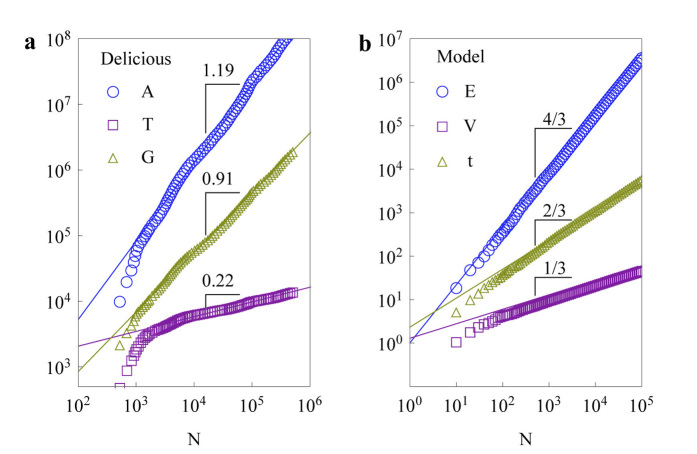
The scaling behaviours of the Delicious community (a) and the model (b). In (a), the scalings represent the cumulative number of activities 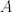 (blue circles), the total time elapsed 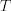 (for convenience of comparison on the plot; filled purple squares), and the cumulative number of tags used by users 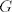 (yellow triangles) versus the cumulative number of users of the Delicious community from Feb 1, 2003 to Nov 8, 2006. The solid lines represent the best fits. In (b), the scalings represent 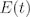 (blue circles), 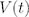 (filled purple squares), and 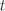 (yellow triangles) versus 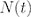 of the basic model 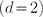 . The solid lines are theoretical predictions of the mean-field approximations (see Supplementary Information Section 1), in all simulations 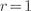 . We also provide the Flickr munity and the APS citation network as other empirical evidence in Supplementary Information Figure S7 and S8.

 This scaling behaviour indicates a densification effect because the growth rate of 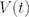 is slower than that of 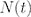 . As a result, the network becomes more and more compact and dense. For 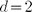 , the scaling exponent is 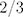 , which is in good agreement with the empirically observed scaling between area and population in cities^27^,^38^. Meanwhile, this scaling also corresponds to the widely observed sub-linear scaling behaviour of diversity (Heap's law) in complex systems if we regard the number of distinct types of entities as the volume in the similarity space^9^ (see Fig. 2 (a)). As a bonus, another scaling to describe the accelerating growth phenomenon can be also analytically obtained: 

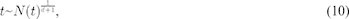

demonstrating that the growth rate of new nodes increases as the size of the network increases 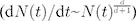 . This phenomenon has been empirically observed in online systems (Fig. 2(a)) and the citation network (see Supplementary Information Figure S8). Our theoretical predictions are in good accordance with the numerical results, as demonstrated in Fig. 2 (b). In addition, we also offer analytical results for the node-degree distributions and for the clustering coefficients, that are determined by the distribution of triangles. (The detailed derivations are provided in Supplementary Information Section 1.4).

### Model Extensions

Although both the super-linear and sub-linear behaviours produced by the basic model are in qualitative agreement with the empirical findings in complex networked systems, a small difference remains between the predicted super-linear scaling exponents 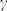 and the real values (For example, the exponent of the super-linear scaling in cities is 1.17, slightly smaller than 4/3). The discrepancy stems from the non-negligible sizes of nodes in real scenarios, such as in cities, which prohibits the birth of an infinite number of nodes in a bounded area. Thus, we incorporate the probability of survival for new nodes even when they are properly matched with some existing nodes to better mimic the real situation. As shown later, this additional feature still allows us to derive analytical results and can produce an adjustable scaling exponent.

#### Crowding effect

A newly generated node can survive with probability 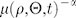 if it matches at least one neighboring node (the distance is smaller than 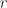 ), where 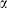 is adjustable and captures the crowding effect. As 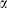 increases, it becomes more difficult for a new node to attach in a dense area. The basic model is recovered if we set 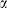 to be zero.

We provide theoretical predictions for the model that incorporates the crowding effect. Suppose that the node density at a given location is 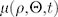 . The number of nodes in 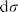 is given by 

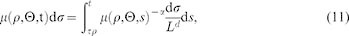

where 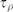 is the time step when 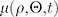 becomes non-zero. Taking the derivative with respect to 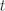 on both sides of Eq. (11), we obtain a differential equation: 

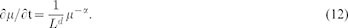

Solving this equation with the initial condition 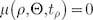 , we obtain 

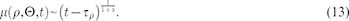

All scalings are produced with adjustable exponents. To be concrete, the scaling between the number of edges and nodes is 

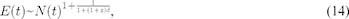

and that between the volume and the number of nodes is 

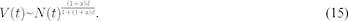

Note that if 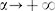 , the exponents in Eqs. (14) and (15) become 1, yielding a linear scaling and a homogenous 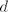 -dimensional regular spatial network. Thus the modified model that incorporates the crowding effects can generally produce scaling behaviours with arbitrary exponent values.

It is interesting to note that the exponents of super-linear scaling and sub-linear scaling in Eqs. (14) and (15) change in different directions when 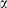 changes. Therefore, observing the sub-linear scaling of diversity may imply the existence of the super-linear scaling of productivity or vice versa, and knowing one exponent may predict another one. That implies diversity phenomenon and productivity in systems are two sides of the same coin. Too fast diversity increase or innovation may slow down the rate of interactions and depress the productivity, a slow and continuous innovation process can accelerate interactions and productivity.

Next, we apply our model to cities. Assume that several cities arise in a 2-dimensional space. The development of these cities is modeled with multiple seeds. We introduce a new rule to naturally mimic the sequential emergence of new seeds. To be concrete, each newly added, surviving node has a probability 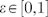 of moving to a random location in the hypercube. By contrast, all existing nodes are not allowed to move. The basic model is recovered for 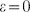 . If 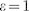 , the model resembles the conventional random geometric graph model^32^. In general, mobile nodes will become new seeds, around which nodes will aggregate, leading to the formation of densely connected local clusters centered on these seeds.

If 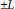 is sufficiently large so that the clusters are isolated, each cluster can grow independently. In this case, the distribution of cluster sizes can be estimated using the Yule-Simon process. Suppose that there are 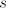 survival nodes and 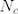 clusters at 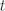 . We use 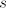 instead of 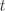 as a new "time" index to facilitate our analysis such that if and only if a new node is attached to existent network, the step 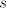 becomes 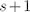 . We denote the volume of the cluster 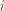 by 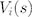 , and the number of nodes in the cluster by 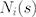 . The probability of a newly added node that attach to cluster 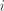 is 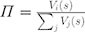 . Because all 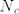 clusters evolve independently, the volume of the 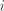 th cluster obeys Eq. (15): 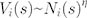 , where 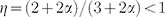 . We thus can write a master equation describing a sub-linear preferential attachment process for all these clusters^39^ (see Supplementary Information Section 4.2): 

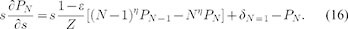

where 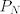 is the fraction of clusters with 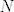 nodes at step 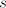 , 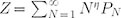 , and 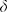 is the Dirac delta function. By solving this equation, we find that the size distribution as 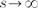 is given by^39^


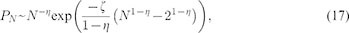

where 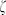 is the solution of the equation 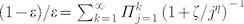 . This distribution resembles a power law if 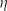 is close to 1. However, 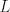 is finite in real situations, leading to certain degree of overlap among clusters and influence on the exponent. The finite-size effect has been explored in detail (see Supplementary Information Section 4.3). The isolation assumption of clusters for deriving analytical results is valid for the scale among cities.

**Figure 3:**
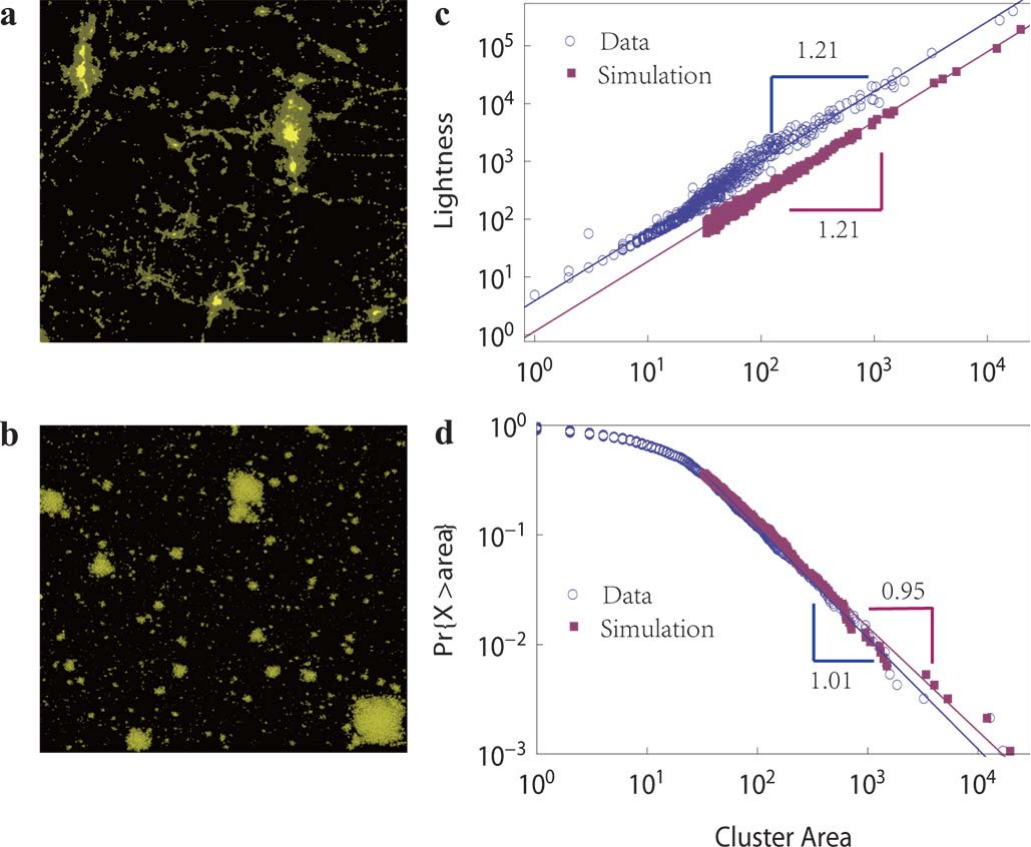
Comparison of modified SCA model with nighttime light data. (a) Satellite image of a nighttime light distribution. We suppose that each connected cluster in the image is a natural city. (b) The clusters grown by the model using the modified rules 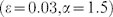 . (c) The scaling between the total area and the total light intensity of these clusters both for the nighttime light image (blue circles) and for the image generated by the model (purple squares). In the model, we take the total number of edges of each cluster to represent its total light intensity. (d) Area distributions of nighttime light clusters (blue circles) and the model's clusters (purple squares). In (c) and (d), only clusters with sizes larger than 33 in simulation are shown for comparison. In all simulations, 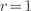 .

If each cluster corresponds to a city, the model by choosing 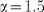 and 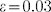 gives rise to the optimal recovery of the empirical results of cities, with scaling exponents^5^ and a power law distribution^30^ that are nearly identical to the real values, as shown in Fig. 3. Moreover, the spatial pattern of aggregation clusters produced by our model is quite similar to those observed in satellite image of nighttime lights in several cities, as shown in Fig. 3 (see Supplementary Information Section 4.1 for more details).

## Discussion

In summary, we developed a growing geometric graph model to uncover the simple underlying mechanisms that account for the super-linear and sub-linear scaling behaviours that are ubiquitously observed in complex networked systems. Our basic model without free parameter is capable of reproducing both categories of scaling behaviours in qualitative agreement with empirical findings, and the scaling exponents are determined exclusively by the dimension of Euclidean space. These results indicate that the SCA plays the primary role in the origins of the scaling behaviours in complex networked systems. However, our model is not limited to Euclidean space. Inserting our SCA mechanism into other spaces, such as hyperbolic space^28^, may offer more general interpretations of generalized scaling behaviours.

To better understand the evolutionary dynamics of real systems, we developed modified models by incorporating crowding effects and multiple seeds with adjustable parameters into the basic model, offering accurate predictions for a variety of empirically observed scaling exponents. For both basic and modified models, we derived analytical results for all important network properties, including the scalings, Zipf distributions, degree distributions and clustering coefficients. The theoretical predictions are in good agreement with the simulated results.

Our approach also offers new insights into the evolution of complex networked systems with respect to the accelerating growth rates of interactions and nodes. Furthermore, our approach provides a deeper understanding of the origins of scaling behaviours in complex networked systems in terms of the trade-off between costs and efficiency, where the former pertains to the interaction density associated with spatial distance and the latter may be measured in terms of topological properties. Taken together, our finding of simple underlying mechanisms that account for the common scaling behaviours in different fields will inspire further effort toward discovering universal rules in the science of complexity.

## Method

### Nighttime light data

The global satellite image of nighttime lights used in our study is collected by the Operational Linescan System (OLS) of the US Air Force Defense Meteorological Satellite Program (DMSP) and archived at NOAA National Geophysical Data Center (NGDC). The image is 30-arc-second grided and spans from -180 to 180 degrees longitude and from -65 to 75 degrees latitude. The digital number ( 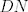 ) values of the nighttime lights range from 1 to 63. In addition, although sunlit data, moonlight, glare, observations containing clouds and lighting features from the aurora are excluded from the DMSP nighttime stable lights dataset, gas flares are not. Therefore, we used the global gas flare map generated by NGDC^40^ to identify and remove gas flares, reducing the possibility of mistaking them for urbanized areas.

The year 2009 was chosen because it was the latest product freely accessible when we first conducted our analysis. For detailed comparison between our model simulation results and nighttime light observations, we narrow our scope down to part of the south central contiguous United States. Using GIS software, the nighttime lights image was re-projected into Lambert conic conformal projection, and a 1000 pixels × 1000 pixels region was extracted from the global image. The upper left corner of the region of interest (ROI) is 113.8 W, 42.2 N, upper right 101.7 W, 43.4 N, lower left 111.7 W, 33.5 N and lower right 100.9 W, 34.5 N. In this region, two lighted pixels were considered as connected if one of them is the Moore neighborhood of the other, and all the connected pixels formed a cluster. Thus we identified 921 clusters in Fig. 3(a). For each cluster, we treated the total number of non-zero pixels as the area of the cluster, and the sum of non-zero pixels values as the total light intensity of the cluster. Then, the scaling between light intensity and area as well as the size distribution of the areas of all clusters were calculated to produce Fig. 3(c) in the main text. More details about nighttime light data can be referred to the Supplementary Information Section 4.1.

### Delicious community data

We compare the scalings of social tagging systems including Delicious and Flickr (see Supplementary Information Section 2) communities to the results of our model. The data can be downloaded freely at http://www.tagora-project.eu/data/. In both systems, users visit certain online resources (pictures on Flickr) and may tag them with certain words. We can consider the semantic space of these tags as the space in which our model is established. Then, the number of distinct tags can be regarded as the total volume occupied by users. And the total number of tagging events can be viewed as interactions happened in the system.

## Author Contributions

JZ conceives and implements the experiments, XL, XW, and LW collect and analyse the empirical data. WW writes the manuscript. All authors reviewed the manuscript.

**Competing Interests** The authors declare that they have no competing financial interests.

## Supplementary Material

Supplementary Information Supporting information
