# Supplementary material for: Scaling behaviours in the growth of networked systems and their geometric origins
Source: Sci Rep. 2015 Apr 29;5:9767. doi: 10.1038/srep09767 (PMC4413893; doi:10.1038/srep09767)
Supplement: Supplementary Information — Supporting information [file srep09767-s1.pdf]

Supplementary Material for

Scaling behaviours in the growth of networked systems and their geometric origins

Jiang Zhang[1*], Xintong Li[1], Xinran Wang[2], Wen-Xu Wang[1*], Lingfei Wu[3,4]

[1] *School of Systems Science, Beijing Normal University*

[2] *College of Resources Science and Technology, Beijing Normal University*

[3] *School of Human Evolution and Social Change, Arizona State University*

[4] *Center for the Study of Institutional Diversity, Arizona State University*

Contents

1 The Mean-Field Analysis of the Basic Model 2

1.1 The linear relation between $R(t)$ and t 2

1.2 Deriving the scaling constants 4

1.3 Simulation tests for the scaling exponent 5

1.4 Other network properties 5

1.4.1 Clustering coefficient 5

1.4.2 Degree distribution 7

2 Additional Empirical Evidence 8

2.1 Flickr 8

2.2 APS citation network 9

3 Model Extension 10

4 Simulation of Nighttime Light Clusters 12

4.1 The nighttime light dataset 12

4.2 The model 13

4.3 Finite size effect 16

5 Supplementary References 18

1 The Mean-Field Analysis of the Basic Model

1.1 The linear relation between $R(t)$ and t

Our mean-field analysis of the basic model starts from a simple and basic relation, $R(t) \sim t$. In this sub section, we present an exact proof of this relation in one dimension and an approximate estimation for $d > 1$ dimensions.

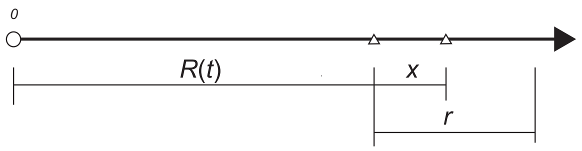

Supplementary Figure S1: Illustration of one-dimensional model. Where $R(t)$ is the radius of the network, the triangle at the position $R(t)$ is the farthest node from the origin 0. If a new node is generated at position $x \in [R(t), R(t) + r]$, it can survive.

In one-dimensional space, we can analyze only one side of the system because of the symmetry of space around the center point. Suppose that at time t, the distance between the farthest node and the center point is $R(t)$ as shown in Supplementary Fig. S1. We know that the distribution of existing nodes lies in the interval $[0, R(t)]$, and is compact, which means that all points in $[0, R(t)]$ are covered by the intervals $[p - r, p + r]$, where p is the coordinate of any existing node. Therefore, $R(t)$ can grow only when a new node is generated in the interval $[R(t), R(t) + r]$. Suppose that the increase of $R(t)$ at t is $\Delta R(t)$. $\Delta R(t)$ is a stochastic variable that can be expressed using the following equations:

$$\Delta R(t) = \begin{cases} x, & \text{if } 0 < x < r \quad \text{(S1a)} \\ 0, & \text{otherwise.} \quad \text{(S1b)} \end{cases}$$

Therefore, the probability density function of $\Delta R(t)$ is

$$f(x) = \begin{cases} 1/L, & \text{if } 0 < x < r \quad \text{(S2a)} \\ 0, & \text{otherwise.} \quad \text{(S2b)} \end{cases}$$

Thus, its expectation value is:

$$\langle \Delta R(t) \rangle = \int_0^r x f(x) \, \mathrm{d}x = \frac{r^2}{L}. \quad \text{(S3)}$$

We can therefore obtain the expectation value of $R(t)$ because

$$\langle R(t) \rangle = \langle \sum_{s=0}^{t} \Delta R(s) \rangle = \sum_{s=0}^{t} \langle \Delta R(s) \rangle = \frac{r^2}{L} t. \quad \text{(S4)}$$

Hence, $R(t)$ is proportional to t. This conclusion is also satisfied for $d > 1$.

In the general case, we define $R(t)$ as the maximum distance from the center to any existing point at time step t. We consider its growth to be $\Delta R(t)$. Because the model is isotropic, the growth in any one direction is similar that in a one-dimensional model. Therefore, if $\Delta R(t) > 0$, any newly generated

node must fall into the volume of the d-dimensional ball of radius r centered on the farthest existing node exclude the $R(t)$ radius d ball centered at the center of the hypercube (the shaded area in Supplementary Fig. S2 represents this region for $d = 2$).

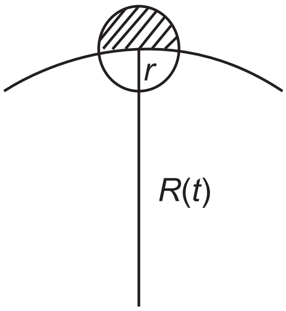

Supplementary Figure S2: Illustration for two-dimensional model. $R(t)$ is the radius of the network, r is the interaction radius. If a new generated node falls into the shaded area, it can survive as well as $R(t)$ will increase.

Because any newly generated nodes will be evenly distributed throughout the hypercube, the probability that one new node will fall into the shaded area is proportional to its d-dimensional volume. When $R(t)$ is very large, this volume is approximately equal to half of the volume of a d-dimensional ball. Therefore, the expectation value of the increase of $R(t)$ is

$$\begin{aligned}\langle \Delta R(t) \rangle &= \frac{\int_{(|\boldsymbol{x}|<r)\wedge(x_1>0)} x_1 \,\mathrm{d}\sigma}{L^d} = \\ &\int_0^r \mathrm{d}\rho \int_0^{\pi/2} \mathrm{d}\theta_1 \int_0^{\pi} \mathrm{d}\theta_2 \cdots \int_0^{2\pi} \mathrm{d}\theta_{d-1} \rho \cos(\theta_1) \rho^{d-1} \sin(\theta_1)^{d-2} \sin(\theta_2)^{d-3} \cdots \sin(\theta_{d-1}) \\ &= \frac{V_{d-1} r^{d+1}}{(d+1)L^d},\end{aligned} \tag{S5}$$

where V_{d-1} is the volume of the $d-1$ - dimensional unit ball, x_1 is the first Cartesian coordinate of a d - dimensional point. Therefore,

$$\langle R(t) \rangle = \frac{V_{d-1} r^{d+1}}{(d+1)L^d} \cdot t. \tag{S6}$$

Equation (S6) not only proves that the radius $R(t)$ is proportional to t but also provides the exact form of the expression for the proportionality constant. This constant scales with r to the power of $d+1$ and L to the power of $-d$. Therefore, when r increases or L decreases, the rate of growth of $R(t)$ will increase. This equation is very important because it can be used to derive other variables. In the main text and the following paragraphs, we use $R(t)$ as an abbreviation for $\langle R(t) \rangle$.

1.2 Deriving the scaling constants

Here, we derive the scaling constants of $V(t)$,t, and $E(t)$. At any given position $(\rho, \mathbf{\Theta})$, the expression for the node density is

$$\mu(\rho, \mathbf{\Theta}, t) = \frac{t - \tau_\rho}{L^d}. \tag{S7}$$

Therefore, the node density at radius ρ is

$$\mu(\rho, t) = \int \mathrm{d}\mathbf{\Theta}\mu(\rho, \mathbf{\Theta}, t)\rho^{d-1} \sin^{d-2}\theta_1 \sin^{d-3}\theta_2 \cdots \sin\theta_{d-2} = \frac{V_d\rho^{d-1}d}{L^d}(t - \tau_\rho) \quad , \tag{S8}$$

where V_d is the volume of a d-dimensional ball. Thus, the total number of nodes is

$$N(t) = \int_0^{R(t)} \mu(\rho, t)\,\mathrm{d}\rho = \frac{V_d}{V_{d-1}r^{d+1}} \cdot R(t)^{d+1}. \tag{S9}$$

Then, because $V(t) = V_d R(t)^d$,

$$V(t) = V_d r^d \left(\frac{V_{d-1}}{V_d}\right)^{\frac{d}{1+d}} N(t)^{\frac{d}{d+1}} \tag{S10}$$

Therefore, the constant of the scaling between $V(t)$ and $N(t)$ is $V_d \left(\frac{r^{1+d}V_{d-1}}{V_d}\right)^{\frac{d}{1+d}}$. We can substitute Eq. (S6) into Eq. (S9) to derive the following:

$$t = (1 + d)\left(\frac{L}{r}\right)^d V_d^{-\frac{1}{d+1}} V_{d-1}^{-\frac{d}{d+1}} N(t)^{\frac{1}{d+1}}. \tag{S11}$$

Thus, the constant of the scaling between t and $N(t)$ is obtained.

Finally, we find the scaling constant between $E(t)$ and $N(t)$. First, we require the exact formula for $v(\rho, \Theta, t)$. We consider a sufficiently small volume $\mathrm{d}\sigma$ in the d-dimensional hypercube, and suppose the size of $\mathrm{d}\sigma$ to be much larger than the constant r. Because nodes in $\mathrm{d}\sigma$ distribute evenly and only nodes pairs separated by distance smaller than r are connected, the total number of links within this infinitesimal volume is

$$\nu\,\mathrm{d}\sigma = \mu\,\mathrm{d}\sigma\mu \int_{|\boldsymbol{x}-\boldsymbol{y}|<r} \mathrm{d}\boldsymbol{x}\,\mathrm{d}\boldsymbol{y} = \frac{1}{2}\mu^2 V_d r^d\,\mathrm{d}\sigma, \tag{S12}$$

where $\boldsymbol{x}, \boldsymbol{y}$ are the coordinates of any two points in $\mathrm{d}\sigma$. A multiplicative factor of $1/2$ arises because the network is undirected. Thus, the link density is

$$\nu(\rho, \Theta, t) = \frac{1}{2}\left(\mu(\rho, \Theta, t)\right)^2 V_d r^d = \frac{1}{2}\frac{(t-\tau)^2}{L^{2d}} V_d r^d \tag{S13}$$

We integrate Eq. (S12) to obtain the total number of links,

$$E(t) = \frac{(d+1)V_d^2}{(d+2)V_{d-1}^2 r^{d+2}} \cdot R(t)^{d+2}. \tag{S14}$$

By substituting the relation between $R(t)$ and $N(t)$ of Eq. (S9), we obtain the following relation between $E(t)$ and $N(t)$:

$$E(t) = \frac{d+1}{d+2} \cdot \left(\frac{V_{d-1}}{V_d}\right)^{-\frac{d}{d+1}} \cdot N(t)^{\frac{d+2}{d+1}}. \tag{S15}$$

All of these relations were tested against numerical simulations, as shown in Fig. 2 in the main text. The results demonstrate that the mean-field approximations can yield exact results.

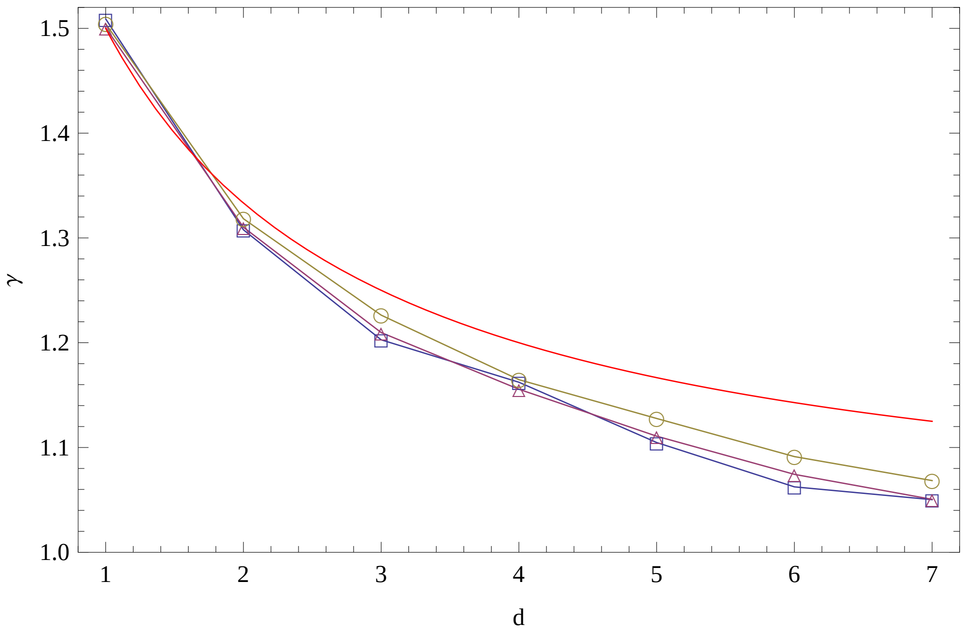

Supplementary Figure S3: The dependence of the exponent $\gamma = (d+2)/(d+1)$ on the dimension d. The blue, purple and yellow joined points were obtained from simulations using different maximum numbers of nodes (different running times): blue corresponds to 10^6 nodes, purple corresponds to 10^5 nodes, and yellow corresponds to 10^4 nodes. The red line represents the theoretical prediction.

1.3 Simulation tests for the scaling exponent

Supplementary Fig. S3 presents comparison of the exponent between the simulations and the theoretical predictions (Eq. (S15)) for various dimensions. The simulated exponents are always smaller than the results of the mean-field approximation, but the discrepancy can be reduced by increasing the running time of the simulation. The reason for this discrepancy is that the mean-field approximation requires that nodes be very dense so that they can be treated as a continuous field.

1.4 Other network properties

Additionally, other network properties, such as the degree distribution and clustering coefficient, can also be derived analytically.

1.4.1 Clustering coefficient

First, we derive the explicit form of the clustering coefficient. According to the definition of the average clustering coefficient for the entire network, it can be understood as the conditional probability that Z is connected to both with X and Y given that X and Y are connected. In our geometric graph model, two

nodes can connect only if the distance between them is smaller than r, and all nodes are distributed evenly throughout the hypercube. Therefore, the computation of the clustering coefficient can be converted into a calculation of volumes.

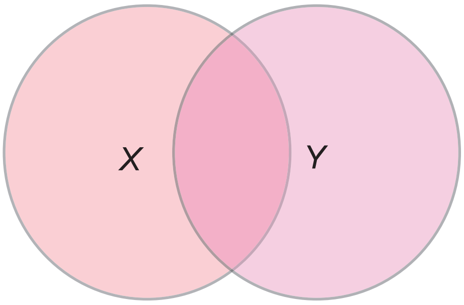

Supplementary Figure S4: Illustration of the calculation for the clustering coefficient

As shown in Supplementary Fig. S4, if we denote the interaction region near node X as the ball X and the interaction region near node Y as Y, then the condition that the two nodes are connected is equivalent to the condition that the overlapped area of the two interaction regions is not empty. If the third node Z is connected to both X and Y, then Z must fall into the overlapped area of X and Y. Therefore, the clustering coefficient C can be calculated using the following formula:

$$C = Pr\{(Z \leftrightarrow X) \wedge (Z \leftrightarrow Y) | (X \leftrightarrow Y)\} = \frac{S_{X \bigcap Y}}{S_X \cdot S_Y} = \frac{3\Gamma(\frac{d+2}{2})}{\sqrt{\pi}\Gamma(\frac{d+1}{2})} \int_0^{\pi/3} \sin^d \theta \, \mathrm{d}\theta, \tag{S16}$$

where $X \leftrightarrow Y$ indicates that X and Y are connected. S_X represents the d-dimensional volume of region X. In Eq. (S16), C is a constant and depends only on the spatial dimension. The theoretical prediction is compared with the results of the computer simulations in Supplementary Fig. S5. It is evident that all results coincide with the theoretical predictions for all dimensions. The results are the same as for the classical random geometric graph.

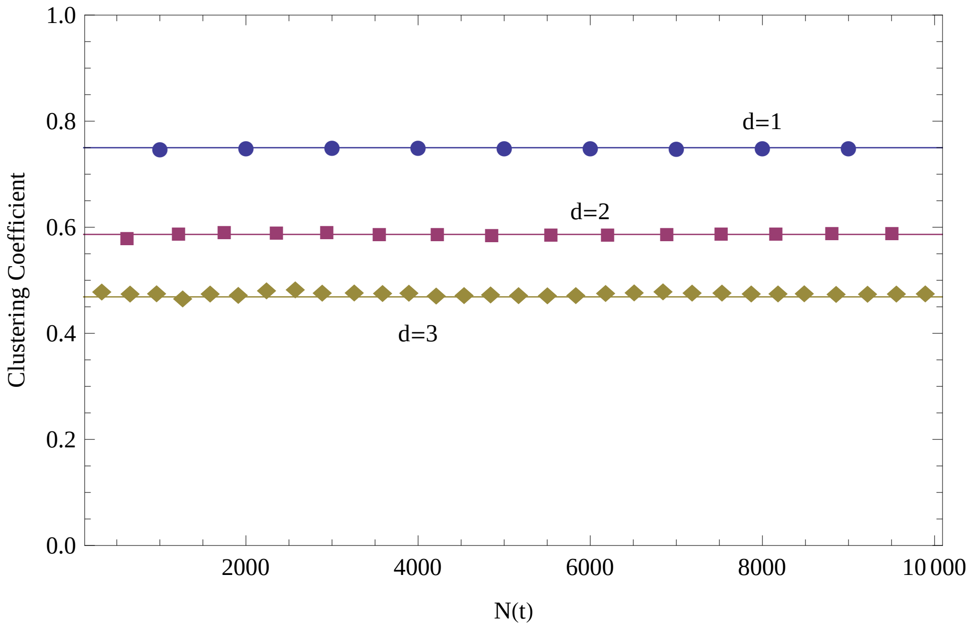

Supplementary Figure S5: Comparison of clustering coefficients between theoretical predictions (solid lines) and computer simulations (points) in $d = 1, 2, 3$ dimensions and for networks of various sizes.

1.4.2 Degree distribution

First, we know that the average degree of one node at radius R at time t is

$$D_R = \int_{|\boldsymbol{x_0}-\boldsymbol{y}|<r} \mu \, \mathrm{d}\boldsymbol{y} \approx \mu V_d r^d = \frac{t-\tau_R}{L^d} V_d r^d. \tag{S17}$$

According to the mean-field approximation, all nodes within the radius R possess degrees larger than D_R; therefore, the cumulative distribution function, i.e., the probability of nodes of degree larger than a given value x, is

$$C_D(x) = Pr\{D > x\} = \frac{\int_0^{R_t - cx} \mu(\rho,t)\,\mathrm{d}\rho}{\int_0^{R_t} \mu(\rho,t)\,\mathrm{d}\rho} = \left(1 - \frac{x}{E_M(t)}\right)^d \left(1 + d\frac{x}{E_M(t)}\right) \quad , \tag{S18}$$

where $c = \frac{V_{d-1} r}{V_d (d+1)}$, and $E_M(t)$ is the maximum degree of the graph at time t. It can be expressed explicitly using the mean-field approximation as follows:

$$E_M(t) = (1+d)\left(\frac{V_{d-1}}{V_d}\right)^{-\frac{d}{d+1}} N(t)^{\frac{1}{d+1}}. \tag{S19}$$

We compare the theoretical result with the simulation result in the upper plot of Supplementary Fig. S6. There is some deviation between the two when x is large. One possible explanation is that only a few nodes possess degrees as large as predicted by the mean-field theory (when t approaches infinity). According to Eq. (S18), the degree distribution density function can be written in the following form:

$$f_D(x) = \frac{1}{N(t)^{1/(d+1)}} g\left(\frac{x}{N(t)^{1/(d+1)}}\right), \tag{S20}$$

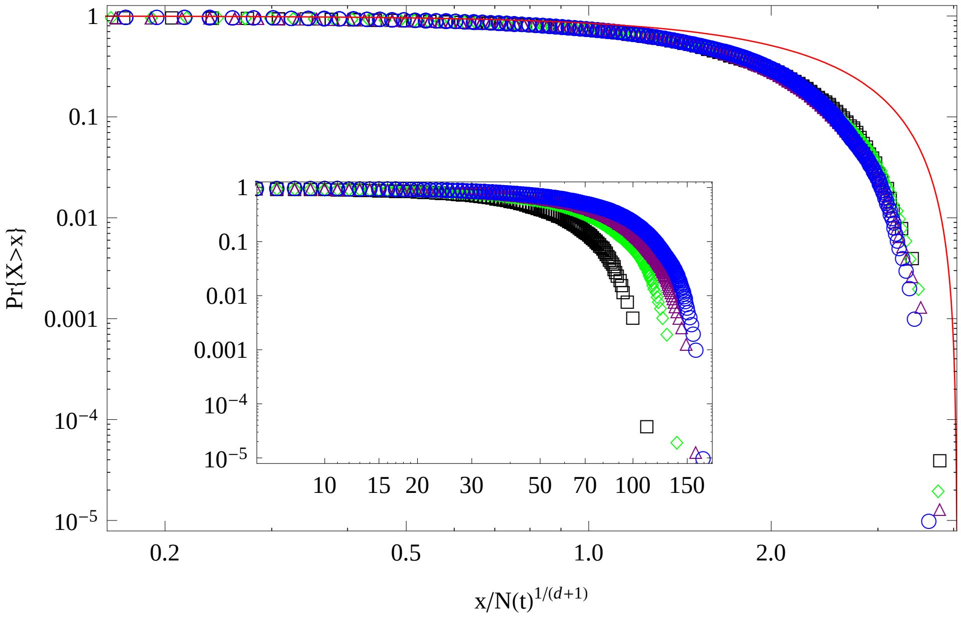

Supplementary Figure S6: Re-scaled size dependent degree distributions of the basic model. The blue circles, purple triangles, green diamonds, and black squares are the degree distribution curves for $N = 10^5, 7.5 \times 10^4, 4 \times 10^4$, and 2.5×10^4 respectively; the inset presents the original results. The solid red line represents the theoretical prediction for the degree distribution (Eq. (S20)).

where

$$g(y) = \frac{d(1+d)y\left(1-\frac{y}{k}\right)^d}{k(k-y)}, \tag{S21}$$

and

$$k = (1+d)\left(\frac{V_{d-1}}{V_d}\right)^{-\frac{d}{d+1}}. \tag{S22}$$

Therefore, the degree distribution is size dependent and also can be re-scaled by the power of the system size $N(t)$ (see Supplementary Fig. S6 (a)). This reflects the self-similarity property of the system, and it has also been observed in other systems [Wu and Zhang(2011)]. Furthermore, the form of the size dependency (Eq. (S20)) also guarantees that the total number of links scales with the number of nodes.

2 Additional Empirical Evidence

2.1 Flickr

We compare the scalings of social tagging systems to the results of our model. In the main text, the Delicious system is studied. Here, we present similar results for another online system, Flickr. The data can be downloaded freely at http://www.tagora-project.eu/data/.

In both systems, users visit certain online resources (pictures on Flickr) and may tag them with certain words. We can consider the semantic space of these tags as the space in which our model is established.

Then, the number of distinct tags can be regarded as the total volume occupied by users.

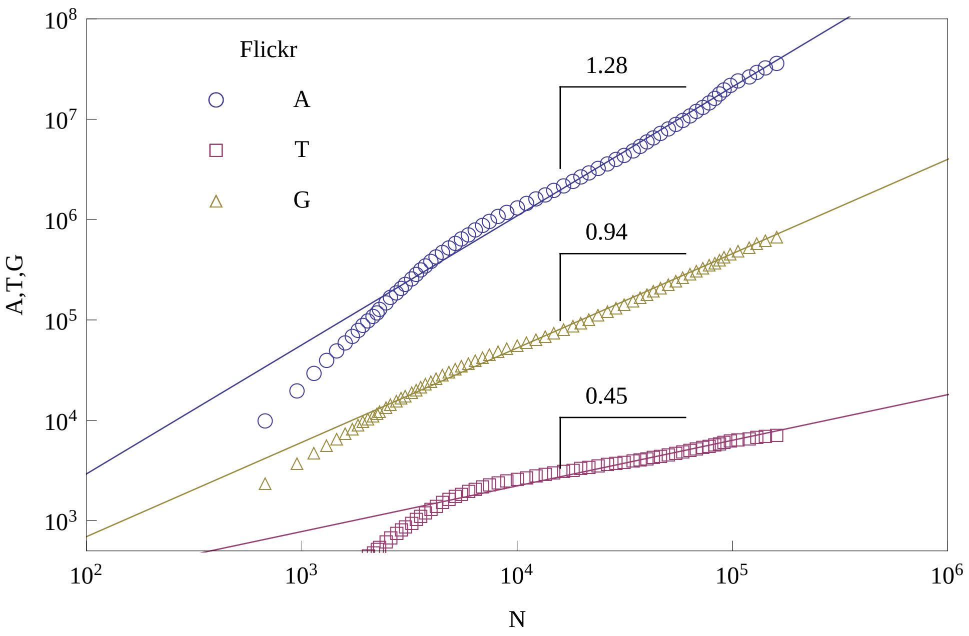

Supplementary Figure S7: The scalings of the Flickr community. The horizontal axis is N, the number of active users. A, G, T represent the total number of activities which have been generated by the active users, the number of tags which have been used, and the time elapsed respectively.

Supplementary Fig. S7 presents the scalings of the Flickr community. In the figure, two distinct stages can be seen with very different growth rates. Initially, the community passed through a phase of relatively slow growth. However, when the size of the system grew to exceed approximately 5000, the rate of growth became very large. The scaling phenomena that we discussed here apply only to the second stage. The delicious community also passed through two such stages, but the separation between them is not apparent because the time window of available data is much longer than that of Flickr.

2.2 APS citation network

As another example, we explore the scaling laws of a citation network based on the APS data set (http://journals.aps.org/datasets). The corpus of Physical Review Letters, Physical Reviews, and Reviews of Modern Physics is comprised of over 450,000 articles and dates back to 1893. Thus, we can construct a citation network from 1893 to 2009. The PACS numbers are used to classify papers since 1977. Therefore, we can calculate the total number of distinct PACS numbers to quantify diversity of the papers. From the empirical data, we find that the super-linear scaling law, i.e., the number of edges versus the number of nodes is of scaling exponent 1.46, and the exponent of the sub-linear scaling law, i.e., time elapsed versus nodes is of scaling exponent 0.24 (see Supplementary Fig. S8). The diversity measured by the number of distinct PACS codes increases suddenly at the beginning because the PACS system is used after 1977. At that time, there are already 48007 nodes in the network. The exponent of

this sub-linear scaling is 0.71.

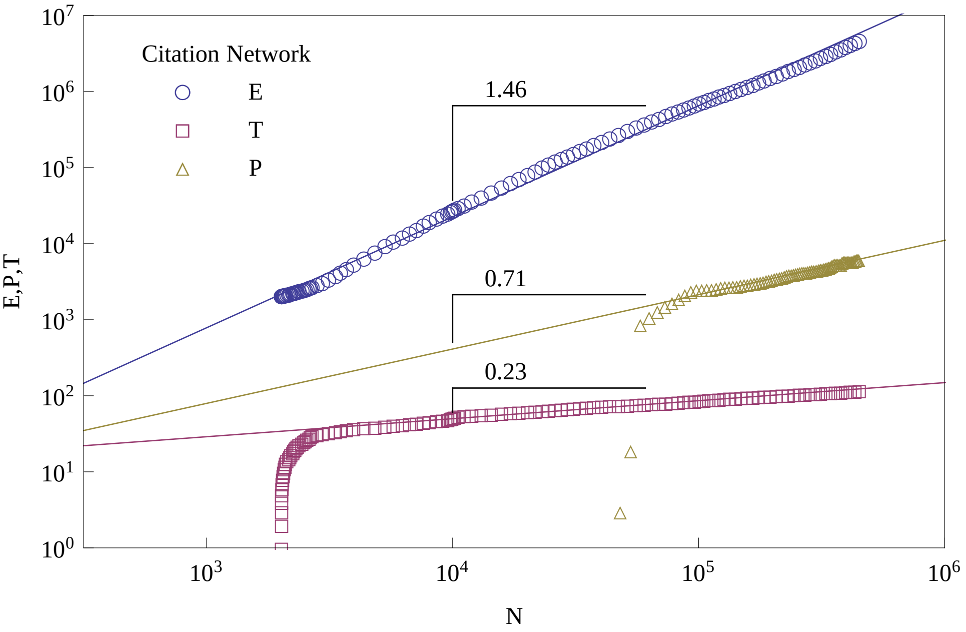

Supplementary Figure S8: The scalings of the APS citation network. N represents the total number of nodes (papers) in the network. E, T and P represent the total number of links (citations) among nodes (E), the total time (years) elapsed (T), and the total number of distinct PACS codes used by those papers (P), respectively.

3 Model Extension

The basic model is very simple, so it can be extended in several respects. As mentioned in the main text, we added the crowding rule to avoid the formation of over-compact graphs. Here, we provide detailed derivation and a discussion of other effects of this extension that are not discussed in the main text.

According to the crowding rule described in the main text, a newly generated node at position $(\rho, \mathbf{\Theta})$ will survive with the following probability:

$$\Pi_s = \begin{cases} \mu^{-\alpha} & \text{if has neighbors} \quad \text{(S23a)} \\ 0 & \text{otherwise.} \quad \text{(S23b)} \end{cases}$$

Therefore, when $\alpha = 0$, the new node will survive with probability 1 if the surrounding node density is nonzero; in other words, we recover the basic model. By contrast, when $\alpha \to \infty$, new nodes will survive in areas of empty space, and we will obtain a random geometric graph with nodes distributed evenly throughout the space. First, we can prove that the relation between $R(t)$ and t does not change when this new rule is incorporated. This is true because the probability that a node falls into the region that can cause the radius of the graph $R(t)$ (which is also defined as the maximum distance of any existing node from the center node) to slightly increase (the shaded area in Supplementary Fig. S2) is the same as in the basic model. Thus the rate of increase of $R(t)$ is also expressed by Eq. (S6).

Next, we will consider the nodes. We can write down a differential equation to describe node density μ:

$$\begin{cases} \partial\mu(\rho, \mathbf{\Theta}, t)/\partial t = \dfrac{1}{L^d}\mu\left(\rho, \mathbf{\Theta}, t\right)^{-\alpha} \\ \mu(\rho, \mathbf{\Theta}, \tau_\rho) = 0 \end{cases} \tag{S24}$$

By solving this equation, we find

$$\mu(\rho, \mathbf{\Theta}, t) = \frac{(t-\tau)^{\frac{1}{1+\alpha}}}{L^d}. \tag{S25}$$

Therefore, the relation between $N(t)$ and $R(t)$ is

$$N(t) \sim R(t)^{d+\frac{1}{1+\alpha}}. \tag{S26}$$

The total number of links between $E(t)$ and $R(t)$ is

$$E(t) \sim R(t)^{d+\frac{2}{1+\alpha}}. \tag{S27}$$

Therefore, we can obtain the scalings with respect to $N(t)$, as mentioned in the main text, and the relation between γ and α, as shown in Eq. (10) of the main text, is

$$\gamma = 1 + \frac{1}{1+(1+\alpha)d}. \tag{S28}$$

We compare these exponents to the results of the numerical experiments in Supplementary Fig. S9. From this figure, it is clear that the mean-field approximation can yield better prediction in $d = 2$ space than in $d = 3$ space. Moreover, as α increases, the deviation between the theoretical prediction and the simulation becomes larger because the nodes become sparser, violating the assumption of the mean-field approximation.

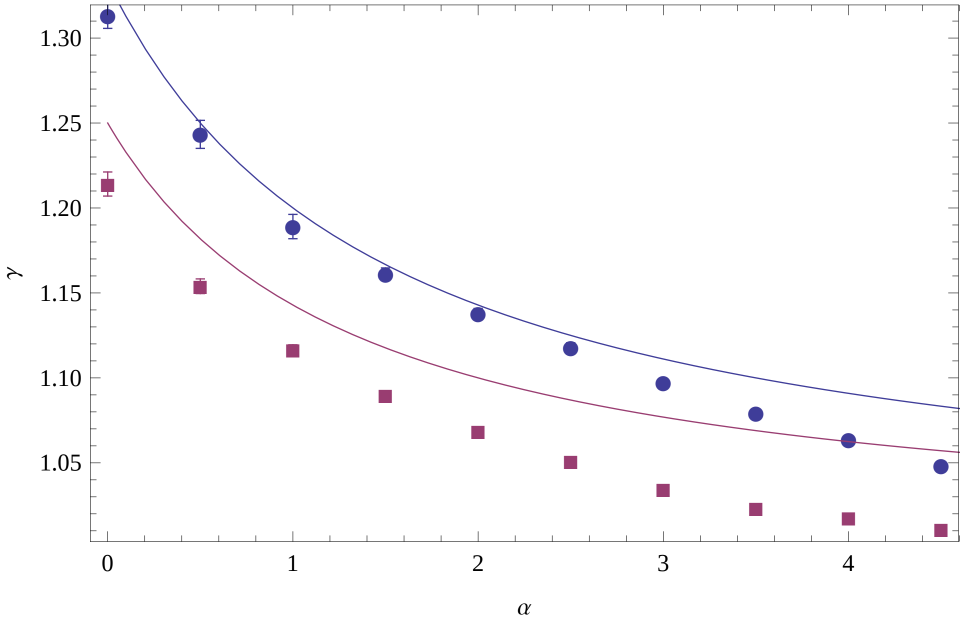

Supplementary Figure S9: Comparison of Eq. (S28) between the mean-field results and the simulation results in $d = 2$ and $d = 3$ spaces. In all simulations, the maximum total number of nodes is 10^5, and $L = 10^{10}$. The blue circles correspond to $d = 2$ and the purple squares correspond to $d = 3$.

4 Simulation of Nighttime Light Clusters

4.1 The nighttime light dataset

The global satellite image of nighttime lights used in our study is collected by the Operational Linescan System (OLS) of the US Air Force Defense Meteorological Satellite Program (DMSP) and archived at NOAA National Geophysical Data Center (NGDC) [NOA(2012)]. The flagship product of this dataset (version 4) is the stable lights. It is an annual cloud-free composite of the average digital brightness values for the detected nighttime lights, altered to remove ephemeral lights and background noise [Elvidge *et al.*(2011)]. The image is 30-arc-second grided and spans from -180 to 180 degrees longitude and from -65 to 75 degrees latitude. The digital number (DN) values of the nighttime lights range from 1 to 63, while 0 represents the identified and eliminated background noise and 255 represents an area where no cloud-free observation has been collected. In addition, although sunlit data, moonlight, glare, observations containing clouds and lighting features from the aurora are excluded from the DMSP nighttime stable lights dataset, gas flares are not. Therefore, we used the global gas flare map generated by NGDC [Elvidge *et al.*(2009)] to identify and remove gas flares, reducing the possibility of mistaking them for urbanized areas.

The year 2009 was chosen because it was the latest product freely accessible when we first conducted our analysis. For detailed comparison between our model simulation results and nighttime light observations, we narrow our scope down to part of the south central contiguous United States, where the

saturated lightness only makes up a negligible proportion so this region suffers less from the well-known saturation problem of DMSP nighttime lights data [Sutton *et al.*(2007)]. Using GIS software, the nighttime lights image was re-projected into Lambert conic conformal projection, and a 1000 pixels x 1000 pixels region was extracted from the global image. The upper left corner of the region of interest (ROI) is 113.8 W, 42.2 N, upper right 101.7 W, 43.4 N, lower left 111.7 W, 33.5 N and lower right 100.9 W, 34.5 N.

In this region, two lighted pixels were considered as connected if one of them is the Moore neighborhood of the other, and all the connected pixels formed a cluster. Thus we identified 921 clusters in Fig. 3(a) in the main text. For each cluster, we treated the total number of non-zero pixels as the area of the cluster, and the sum of non-zero pixels' values as the total light intensity of the cluster. Then, the scaling behaviour between light intensity and area as well as the size distribution of the areas of all clusters were calculated to produce Fig. 3(c) in the main text.

4.2 The model

In the main text, we introduced a parameter ϵ to simulate cluster formation. We provide the details of its implementation in this subsection.

In the simulation, we set a rectangular region of width 1000 and height 1000 which are equal to the width and height of the nighttime light image, respectively, in units of pixels. Initially, there are no nodes in the rectangle. In every time step, one node is added to the region, and the survival of that node is determined in accordance with the crowding rule with parameter α. If the node survives, it may become a seed with a probability of ϵ. In each time step, we identify all nodes that are connected by edges as clusters. The nodes are continuously generated, one by one, until the number of clusters larger than 33 in size(the starting point of the power-law tail in the empirical data) is equal to 335, the number of clusters in the power-law tail of the empirical data. Then, we calculate the scaling behaviour between area and light intensity and the size distribution of the clusters.

The clusters created at earlier times may have more chances to gain new nodes, and thus, they may become very large. By contrast, some newly born clusters may be very small, and some clusters may also encounter each other and merge. We locate each node on the lattice by means of the integer parts of its coordinates. Then, the area of each cluster is calculated as the total number of distinct lattices occupied by all nodes in that cluster. The total light intensity is calculated as the total degrees (number of edges) of all the nodes in one cluster because we assume that the light intensity of a cluster is proportional to the total number of interactions. Then, by tuning $\alpha = 1.5$ and $\epsilon = 0.03$, we can generate exponents of the scaling between area and light intensity and a size distribution that are similar to those determined from the empirical data.

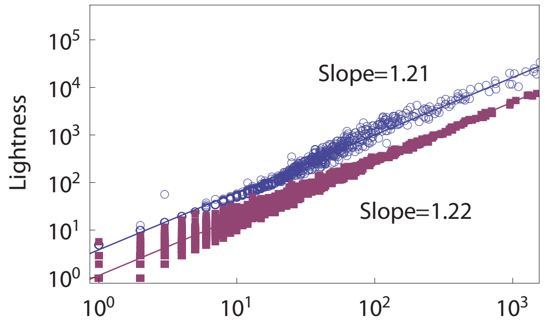

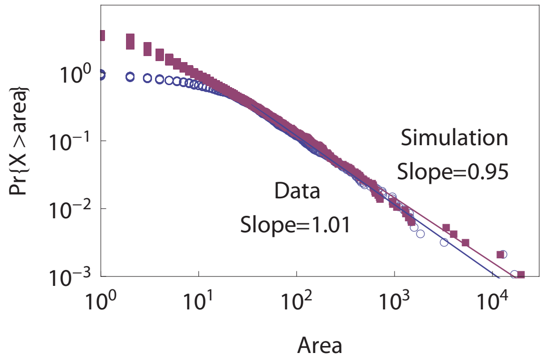

Supplementary Figure S10: The original plots of scaling behaviour and area distribution for simulations and nighttime light data.

The scaling behaviour plot and the cumulative distribution of areas are presented in Supplementary Fig. S10. However, Fig. 3 in the main text shows that the cluster size distribution of the satellite data does not follow a strict power-law. The power-law tail starts at a size of 33. However, our model can generate a power law distribution because of the underlying Yule-Simon mechanism. Therefore, in the main text, we plot only clusters with areas larger than 33. The entire plot with all clusters included is presented in Supplementary Fig. S10. We are aware that the scaling law for small clusters is not very clear because of large fluctuations. We believe that with some small changes to its implementation, our model will be able to generate better statistics that will be comparable to the empirical data. This task will be left for future studies.

In the main text, we mention that the dynamics of the change in cluster sizes with the number of nodes can be approximated as a Yule-Simon process with an exponent smaller than 1 because the area of the region is very large. We present the analysis that supports this statement in detail here.

If $L \to \infty$, then the clusters may be isolated from each other. Therefore, the dynamics of each cluster can be viewed as an independent random process.

Suppose that at time t, there are s existing nodes and $N_C(t)$ clusters. We let $M_N(t)$ be the total number of clusters with N nodes, and $P_N(t) = M_N(t)/N_C(t)$ is then the fraction of such clusters among all clusters. We use s as the time index such that in a single step, only one node is added to either join one of the existing N_C clusters or, with probability ϵ, form a new cluster. Therefore, we can express

the dynamical equation of $M_N(s)$ as follows:

$$\frac{\partial M_N(s)}{\partial s} = \frac{1-\epsilon}{bZ}\left[b(N-1)^{\eta} M_{N-1}(s) - bN^{\eta} M_N(s)\right] + \epsilon \cdot \delta_{N=1}, \tag{S29}$$

where $Z = \sum_{N=1}^{\infty} N^{\eta} M_N(s)$, b is the scaling behaviour constant between the area $V(t)$ and the number of nodes $N(t)$, and $\eta = (2+2\alpha)/(3+2\alpha) < 1$. According to

$$M_N(s) = N_C(s) \cdot P_N(s). \tag{S30}$$

we find that

$$\frac{\partial N_C(s)}{\partial s} P_N(s) + N_C(s) \frac{\partial P_N(s)}{\partial s} = N_C(s) \frac{1-\epsilon}{Z}\left[(N-1)^{\eta} P_{N-1}(s) - N^{\eta} P_N(s)\right] + \epsilon \delta_{N=1}, \tag{S31}$$

and because,

$$N_C(s) = \epsilon s, \tag{S32}$$

we find that

$$\epsilon P_N(s) + \epsilon s \frac{\partial P_N(s)}{\partial s} = \epsilon s \frac{1-\epsilon}{Z}\left[(N-1)^{\eta} P_{N-1}(s) - N^{\eta} P_N(s)\right] + \epsilon \delta_{N=1}. \tag{S33}$$

Thus, we can derive Eq. (12) in the main text. We are interested in the stable state, so we let $s \to \infty$; then, $\frac{\partial P_N(s)}{\partial s} = 0$, and we obtain

$$P_N(s) = s \frac{1-\epsilon}{Z}\left[(N-1)^{\eta} P_{N-1}(s) - N^{\eta} P_N(s)\right] + \epsilon \delta_{N=1}. \tag{S34}$$

When $s \to \infty$, we can set $Z \sim s$ because we know that $N^0 < N^{\eta} < N^1$, and thus, $s = \sum_N N^0 M_N(s) < \sum_N N^{\eta} M_N(s) < \sum_N N^1 M_N(s) = N_C = \epsilon s \sim s$. Therefore, $\sum_N N^{\eta} M_N(s) \sim s$. We introduce an undetermined parameter ζ, and let

$$Z = (1-\epsilon) \cdot \zeta \cdot s, \tag{S35}$$

and insert it into Eq. (S34):

$$P_N = \frac{1}{\zeta}\left[(N-1)^{\eta} P_{N-1} - N^{\eta} P_N\right] + \epsilon \delta_{N=1}. \tag{S36}$$

We can solve P_N using this equation,

$$P_N = \zeta N^{-\eta} \prod_{j=1}^{N} \left(1 + \frac{\zeta}{j^{\eta}}\right)^{-1} \tag{S37}$$

This quantity can be approximated as

$$P_N = \zeta N^{-\eta} \exp\left[-\zeta \left(\frac{N^{1-\eta} - 2^{1-\eta}}{1-\eta}\right)\right], \tag{S38}$$

where, ζ can be obtained using the following equation

$$\frac{1-\epsilon}{\epsilon} = \sum_{N=1}^{\infty} \prod_{j=1}^{N} (1 + \frac{\zeta}{j^{\eta}})^{-1}. \tag{S39}$$

And we insert $V = bN^{\eta}$ into Eq. (S38) to obtain the probability density function of areas as,

$$P_V = \frac{\zeta}{b\eta}(\frac{V}{b})^{\frac{1}{\eta}-2} \exp\left[-\zeta\left(\frac{(V/b)^{\frac{1-\eta}{\eta}} - 2^{1-\eta}}{1-\eta}\right)\right]. \quad \text{(S40)}$$

It is hard to obtain the explicit form of this distribution because ζ is difficult to calculate. Therefore, we show the numerical result of Eq. (S40). A power law function with exponent dependent on ϵ is obtained (see Fig. S11).

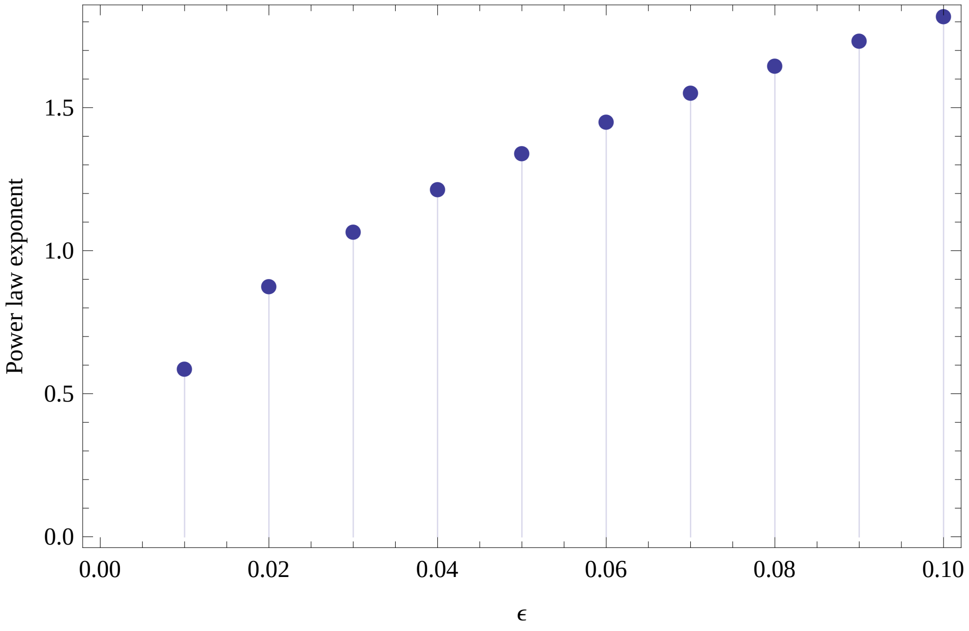

Supplementary Figure S11: The exponent of the cumulative size distribution as a function of ϵ in the numeric results of Eq. (S40) by setting $\alpha = 1.5$.

4.3 Finite size effect

In real situations, L is finite, leading to possible collision of some clusters. If this occurs, the analytical results based on the assumption of isolated clusters may be violated. Therefore, we explore the influence of the crossover of clusters on the scaling exponents by carrying out simulations with continuous generation of new nodes for L = 200, 300, 500, respectively. Without loss of generality, we set $\epsilon = 0.03$ and $\alpha = 1.5$. The results are shown in Supplementary Fig. S12, S14, and S13.

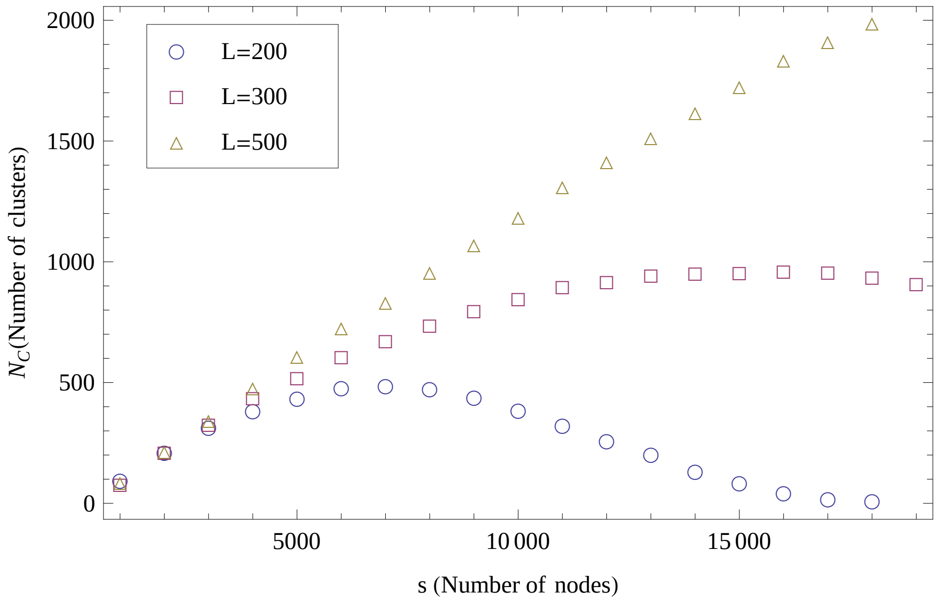

Supplementary Figure S12: The number of clusters N_C as a function of the total number of nodes s for different values of L.

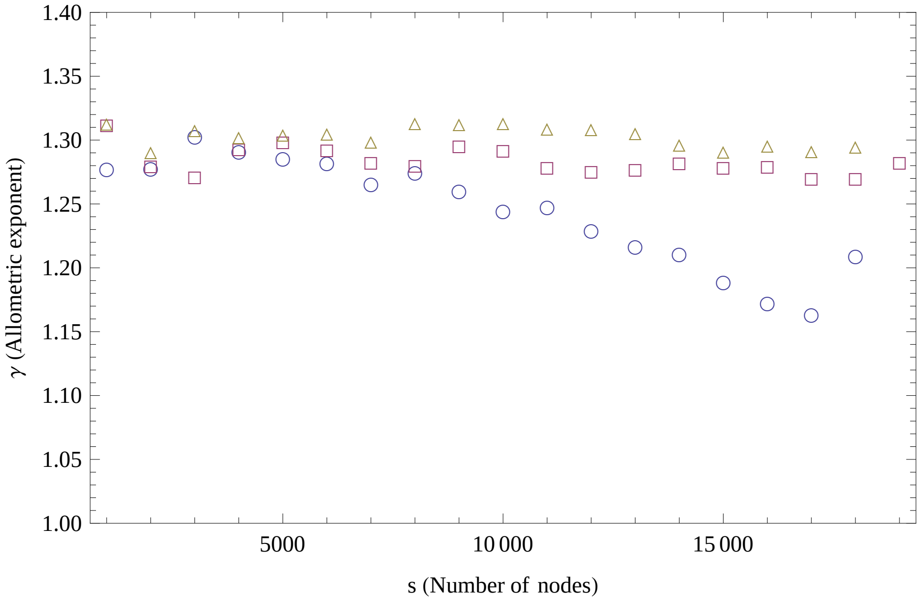

Supplementary Figure S13: The scaling exponent γ as a function of the total number of nodes s (Blue circles for $L = 200$, pink squares for $L = 300$, and yellow diamonds for $L = 500$).

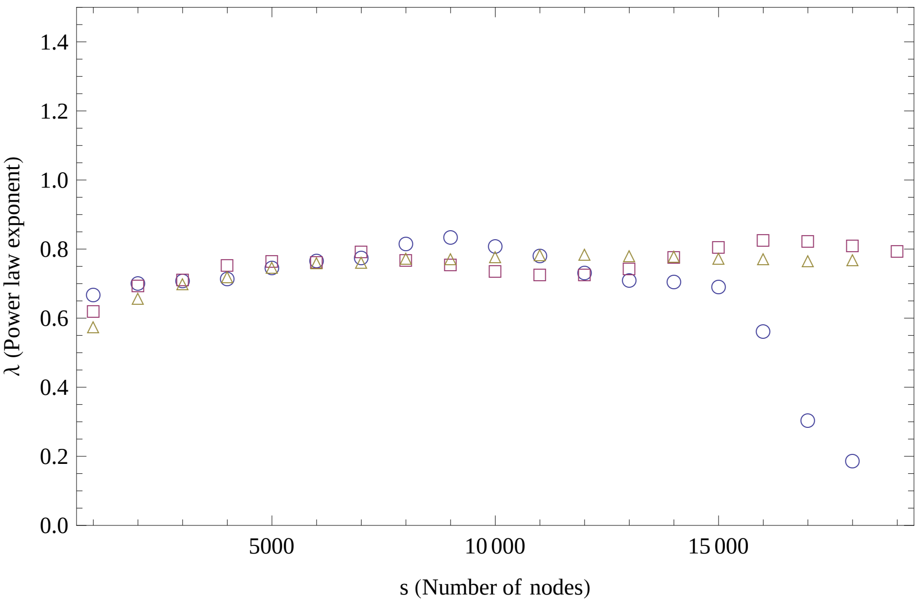

Supplementary Figure S14: The power-law exponent of the cumulative size distribution as a function of the total number of nodes s (Blue circles for $L = 200$, pink squares for $L = 300$, and yellow diamonds for $L = 500$).

We find that for finite L, as the number of nodes s increases, crossovers take place more frequently (Supplementary Fig. S12) until only one super cluster remains for $L = 200$. We see that the scaling exponent almost remains unchanged for a wide range of s. For very large s, the scaling exponent slowly decreases, resulting from crossovers (Supplementary Fig. S13). This phenomenon becomes more obvious for small L. Supplementary Fig. S14 shows that the power-law exponent of cumulative size distribution also remains stable until the finite size effect works. Take together, these results indicate that the scaling exponents are insensitive to the variation of the number of nodes and crossovers when L is large, accounting for the validity of the approximation of isolated clusters for theoretically predicting the scaling exponents with respect to finite size of the space and a large number of nodes. In addition, our simulation results indicate that the scaling exponents of real cities may decrease as the sizes of cities increase, because of the finite geographical space, in which cities are embedded, as shown in Supplementary Fig. S13 and Supplementary Fig. S14.

5 Supplementary References

[Wu and Zhang(2011)] L. Wu and J. Zhang, Phys. Rev. E, **84**, 026113 (2011).

[NOA(2012)] "NOAA national geophysical data center, version 4 DMSP-OLS nighttime lights time series, http://www.ngdc.noaa.gov/dmsp/downloadv4composites.html," (2012).

[Elvidge *et al.*(2011)] C. Elvidge, P. Sutton, K. Baugh, D. Ziskin, T. Ghosh, and S. Anderson, *AGU Fall Meeting Abstracts*, **1**, 03 (2011).

[Elvidge *et al.*(2009)] C. D. Elvidge, D. Ziskin, K. E. Baugh, B. T. Tuttle, T. Ghosh, D. W. Pack, E. H. Erwin, and M. Zhizhin, Energies, **2**, 595 (2009).

[Sutton *et al.*(2007)] P. C. Sutton, C. D. Elvidge, and T. Ghosh, International Journal of Ecological Economics & Statistics, **8(7)**, 5 (2007).
